# Editorial: Recent advances in ameloblastoma research and management in dogs

**DOI:** 10.3389/fvets.2024.1485342

**Published:** 2024-09-24

**Authors:** Santiago Peralta, Boaz Arzi

**Affiliations:** ^1^Department of Clinical Sciences, College of Veterinary Medicine, Cornell University, Ithaca, NY, United States; ^2^Department of Surgical and Radiological Sciences, School of Veterinary Medicine, University of California, Davis, Davis, CA, United States

**Keywords:** oral tumor, canine acanthomatous ameloblastoma, reconstruction, immunohistochemistry, surgical margins, HRAS gene mutation

Canine acanthomatous ameloblastoma (CAA) is currently the most widely used term for a well-recognized, relatively prevalent, and locally aggressive oral tumor of epithelial origin. Despite its clinical importance, the biological mechanisms of tumor initiation and progression remained largely unexplored for decades, and most of the diagnostic and therapeutic principles have historically been based on heuristics. Notable examples include assigning a cell-type of origin (i.e., rests of Malassez, rests of Serres) without conclusive scientific evidence, and the adoption of confusing clinical and histological nomenclature that constantly evolves due to diverging expert opinions, or that is influenced by changes in human medical tumor classification that may or not correspond. Similarly, proposed surgical, radiation or chemotherapeutic strategies have traditionally lacked a robust evidence base, and in many ways may be regarded as perpetuated dogma. Recent studies that leverage genomic and tissue engineering technologies, and apply more rigorous study designs, have started to change our understanding of CAA, and are helping redefine ways in which tumors are diagnosed, classified, and treated. In this Research Topic we include a review article that examines proper surgical margins in CAA, a case series that describes one institution's experience with tissue engineering solutions for reconstruction of mandibles following mandibulectomy to remove CAA, and a controlled study that validates a novel immunohistochemical diagnostic solution based on the mutational profile of CAA. These articles are illustrative examples of current scientific trends that are already changing the state-of-the-art.

In a comprehensive review article, Goldschmidt critically appraises the veterinary literature surrounding adequate surgical margins for CAA excision, and how this might affect patient outcomes. She notes that marginal excision of CAA may result in a high rate of recurrence, whereas surgical margins of 10 and 20 mm are associated with very low recurrence rates. However, wide-margin excisions may result in unacceptable patient morbidity, and some evidence is provided that suggests that excellent outcomes may be achieved with less aggressive interventions. She also notes that such surgical technicalities will remain controversial as long as they are not informed by solidly designed studies and invites others to generate additional and more robust clinical data to help guide future surgical standards.

In their case series, Tsugawa et al. demonstrated successful CAA excision in a series of 11 dogs and share the group's experience using the regenerative approach of a titanium locking plate and compression resistant matrix (CRM) infused with rhBMP2 for the immediate or delayed reconstruction following mandibulectomy. In all 11 dogs that received the reconstructive procedure, surgical planning included conventional computed tomography (CT), with and without contrast. In a subset of cases, 3D-printed models were used for surgical planning. The manuscript described that tumor-free surgical margins were achieved in all dogs following mandibulectomy. The study included long-term clinical and diagnostic imaging follow-up (mean, 23.1 months). It was demonstrated that at 2 weeks postoperatively hard tissue formed at the defect site. Follow-up CT imaging revealed that the regenerated bone had heterogeneous appearance and was bridging the gap defect. That regenerated bone remodeled over 3–6 months to become bone of a similar size, shape, and trabecular pattern as native bone. Evaluation of the histological features of the regenerated bone revealed that it was supportive of the clinical and imaging findings of normal remodeled bone. Clinically, all dogs in that series returned to a normal function and rapidly resumed eating and drinking and exhibited normal occlusion. The authors noted minor complications in three dogs such as wound dehiscence in one dog and self-limiting exuberant bone formation in two dogs. It was concluded that mandibular reconstruction using a regenerative approach is safe, feasible and results in restoration of mandibular contour in dogs following segmental and bilateral rostral mandibulectomy for oral tumors such as CAA.

In a controlled study, Peralta et al. demonstrate that a commercially available anti-RAS p.Q61R antibody (SP174), originally designed for mouse and human immunohistochemistry (IHC) applications, detects the mutated protein in formalin-fixed paraffin-embedded CAA tissues with a 100% specificity and 100% sensitivity. Given that previous work (1) by the same group demonstrated that more than 90% of CAA tumors harbor an *HRAS* p.Q61R mutation, and that IHC assays are widely available in diagnostic laboratories, this represents an economically, technically and logistically feasible way to rapidly and reliably distinguish between CAA and other oral tumors that do not harbor this mutation, including oral squamous cell carcinoma (OSCC). The latter is of relevance given that CAA and OSCC are often times misdiagnosed due to overlapping histological features despite major differences in clinical and biological behavior including OSCC's more aggressive and invasive local behavior, metastatic potential and significantly higher proliferation activity (2).

As these articles make clear, investigations that take advantage of biomedical technologies and apply comparative, analytical and rigorously designed methodologies, are more likely to result in full translational cycles. We hope that the articles contained in this Research Topic will motivate others to continue to investigate CAA and other oral tumors in dogs in ways that will help move quality-of-veterinary-care forward, in the context of One Health and optimized outcomes.
